# Efficacy and Safety of PARP Inhibitor Combination Therapy in Recurrent Ovarian Cancer: A Systematic Review and Meta-Analysis

**DOI:** 10.3389/fonc.2021.638295

**Published:** 2021-08-13

**Authors:** Ning Ren, Leyin Zhang, Jieru Yu, Siqi Guan, Xinyang Dai, Leitao Sun, Minli Ying

**Affiliations:** ^1^The First School of Clinical Medicine, Zhejiang Chinese Medical University, Hangzhou, China; ^2^School of Basic Medical Sciences, Zhejiang Chinese Medical University, Hangzhou, China; ^3^Department of Traditional Chinese Medicine, Beijing Obstetrics and Gynecology Hospital, Capital Medical University, Beijing, China; ^4^Department of Medical Oncology, The First Affiliated Hospital of Zhejiang Chinese Medical University (Zhejiang Provincial Hospital of Traditional Chinese Medicine), Hangzhou, China; ^5^Department of Gynaecology, The First Affiliated Hospital of Zhejiang Chinese Medical University (Zhejiang Provincial Hospital of Traditional Chinese Medicine), Hangzhou, China

**Keywords:** PARP inhibitor, combination therapy, monotherapy, efficacy, safety, meta-analysis

## Abstract

**Objectives:**

Though it is known to all that PARP inhibitors (PARPis) are effective when used as maintenance alone for women with recurrent ovarian cancer (ROC), little is known about whether using them in combination with other drugs would contribute to a better efficacy. We performed a systematic review and meta-analysis to explore the efficacy and safety of PARPi combination therapy compared with monotherapy.

**Materials and Methods:**

We searched for randomized controlled trials (RCTs) that offered the date we needed in PubMed, Embase, Cochrane, and major conference. Data extraction and processing were completed by three investigators to compare OS, PFS, and ORR both in intervention and in control subset. Then, we calculated the pooled RR and 95% CI of all-grade and high-grade adverse effects to study its safety. And we evaluated the within-study heterogeneity by using subgroup and sensitivity analysis.

**Results and Conclusion:**

A total of three eligible RCTs covering 343 women were included. In PFS analysis, PARP inhibitor (PARPi) combination therapy can significantly improve PFS for women with ROC when compared with the controls (HR: 0.46, 95% CI: 0.35 to 0.59), especially for those with mutated BRCA (HR: 0.29, 95% CI: 0.19 to 0.45). And in OS analysis, combination therapy is not inferior to monotherapy (HR: 0.90, 95% CI: 0.50 to 1.61). As for ORR, the effectiveness of combination therapy and monotherapy was almost the same (RR: 1.04, 95% CI: 0.82 to 1.31). Additionally, combination therapy seldom causes more adverse events, both in all-grade and in high grade.

**Systematic Review Registration:**

https://www.crd.york.ac.uk/PROSPERO/, International Prospective Register of Systematic Reviews (PROSPERO) (identifier, CRD42018109933).

## Introduction

Ovarian cancer (OC), with poor prognosis, is one of the most prevalent gynecologic malignancies. Each year, about 295,414 people are diagnosed with OC all over the world, and 184,799 patients die due to this disease with the 1-year mortality rate up to 63% (1). To remove the tumor and make a definite diagnosis and staging, surgical treatment is the first choice for early OC. While cytoreductive surgery combined with platinum-based chemotherapy is usually used for advanced OC (2), despite short-time effect, there are still about 70% of patients suffering from the recurrence after first-line treatment (3), which seriously affects one’s survival time (4).

If the disease recurs 6 months or longer after first-line treatment, further platinum-based therapy and debulking surgery are widely used at first relapse. Moreover, chemotherapy will produce chemotherapy-related side effects in patients, and those patients will consequently become treatment-resistant, succumbing to disease (5). Therefore, since cumulative myelosuppression, neurotoxicity, and allergy to platinum-based therapy can be limiting factors in patients receiving multiple lines of treatment (6), new effective therapeutic strategies are needed.

After various trials for the need of other treatment methods, an active therapeutic target for combination treatment was found to be the DNA damage response pathway, such as Poly(ADP-ribose) polymerase (PARP). PARP inhibitor (PARPi) has been proven to cause DNA damage *via* catalytic inhibition of the PARP enzyme and trapping of DNA-PARP complexes, which results in synthetic lethality in cells deficient in homologous recombination repair, and consequently strengthen the use of killing tumor cells (7, 8). This process is called “PARP trapping.” The six available PARPis in the clinic are the olaparib, rucaparib, niraparib, talazoparib, pamiparib, and veliparib, among which the olaparib, rucaparib, and niraparib have been approved by the US Food and Drug Administration (FDA) and/or the European Medicines Agency (EMA).

Nowadays, many patients with platinum-sensitive, recurrent OC (ROC) benefit from PARPi maintenance therapy for this mechanism (9), but the efficacy of PARPi monotherapy is also of limited therapeutic effect for not the vast majority of women with ROC could benefit well from it, which limits its application. Given this background, we suspect that combination therapy can make up for the deficiency of monotherapy and will work in a shorter period of time compared with monotherapy, which may work by synergism.

Besides, few people choose to study the application value of PARPi combination to refine their use, and combination therapy might be prone to adverse events *versus* monotherapy. So we plan to perform a systematic review and meta-analysis to compare the validity, superiority, and drug safety of combination therapy with monotherapy based on the results of survival analysis, overall response rate (ORR), safety, and the screening of the most suitable population.

## Methods

### Search Strategy

Three investigators independently retrieved all the related studies in the databases including PubMed, Cochrane, and Embase for the most update randomized controlled trials (RCTs) until November 25, 2020, to explore PARPi combination therapy for ROC compared with monotherapy on its clinical benefit and risk (**Supplementary Method 1**). Moreover, we obtained the data sources from the abstract and presentations recorded in some annual meeting, symposium, or congress such as ASCO, ESMO, ESGO, and so on to ensure that the relevant minutes were not overlooked. Only the most complete and cutting-edge trials were included when duplicate publications were identified.

### Selection Criteria

Our meta-analysis had been registered at International Prospective Register of Systematic Reviews (number: CRD42018109933) and is supposed to meet the requirement of Preferred Reporting Items for Systematic Reviews and Meta-Analyses (PRISMA). Studies meeting all of the following criteria were included: (1) Randomized controlled phase II or III trials in women who were histologically or cytologically diagnosed with OC before and had relapsed after initial cure; (2) In intervention group, women were treated with PARPi in combination with chemotherapy, other targeted agents, or immune-oncology agents and so on; (3) Patients were treated with control regimen including chemotherapy, other targeted agents, or immune-oncology agents and so on in monotherapy; (4) Studies own data available for hazard ratio (HR) and 95% credible interval (CI) of progression‐free survival (PFS) or overall survival (OS). In the meantime, articles were directly eliminated in the following cases: (1) Case report, review, meta-analysis, or only laboratory research; (2) Only in the form of meeting abstracts without available data for analysis; (3) Clinical study that was not based on the RCTs; (4) Studies that were phase I or retrospective.

Three independent investigators looked through each article by their titles and abstracts to pick up potentially relevant articles meeting the predefined inclusion criteria. Then, they carefully read the full text of the remaining articles, which were included from previous screening, to select the suitable ones. All disagreements about selection between investigators were discussed and resolved by all investigators.

### Risk of Methodological Bias Assessment

Two investigators independently evaluated the risk of bias in included studies and assessed it as low, unclear, or high risk of bias by applying the Cochrane evaluation handbook of RCTs (5.1.0), which includes the following characteristics covering randomization sequence generation (selection bias), allocation concealment (selection bias), blinding of participants and personal (performance bias), blinding of outcome assessment (detection bias), incomplete outcome data (attrition bias), selective reporting (reporting bias), and other biases.

### Data Extraction

Three investigators independently extracted the individual data and recorded them in a standard form. The following information, which was valid and complete, was acquired from each included study on the basis of eligibility criteria by following under PRISMA: (1) Study baseline characteristics: first author, publication time, masking, line, pathology, follow-up, phase, regimen, and group; (2) Study population: number in each arm, median age, age range; (3) Study outcomes: HR with 95% CI for OS and PFS. We also calculated HR with 95% CIs for ORR and relative risk (RR) with 95% CI for safety analysis built on the data obtained from all included studies. We also reviewed each clinical trial’s supplement.

### Statistical Analysis

All data were expressed as the combination of HR or RR and 95% CI, and P<0.05 was considered statistically significant. STATA 15 was used to perform statistical analyses including pooling the data and producing the forest plots. I² test was used to assess the between-study heterogeneity, which estimated the percentage of total variability across all studies. The data would be calculated through a random‐effects model once the test showed I² > 50% or P < 0.10. Besides, I² regarded an estimated value applied three fixed knots at 25, 50, and 75% as an indicator of mild, moderate, and high heterogeneity. Otherwise, we used a fixed‐effects model to pool effect size. In order to deeply explore the heterogeneity and its potential influence, we also performed subgroup analysis. Sensitivity analysis, which examined the robustness of included trials to different aspects, was performed by step-wise removal of single study.

## Results

### Identification and Selection

**Figure 1:**
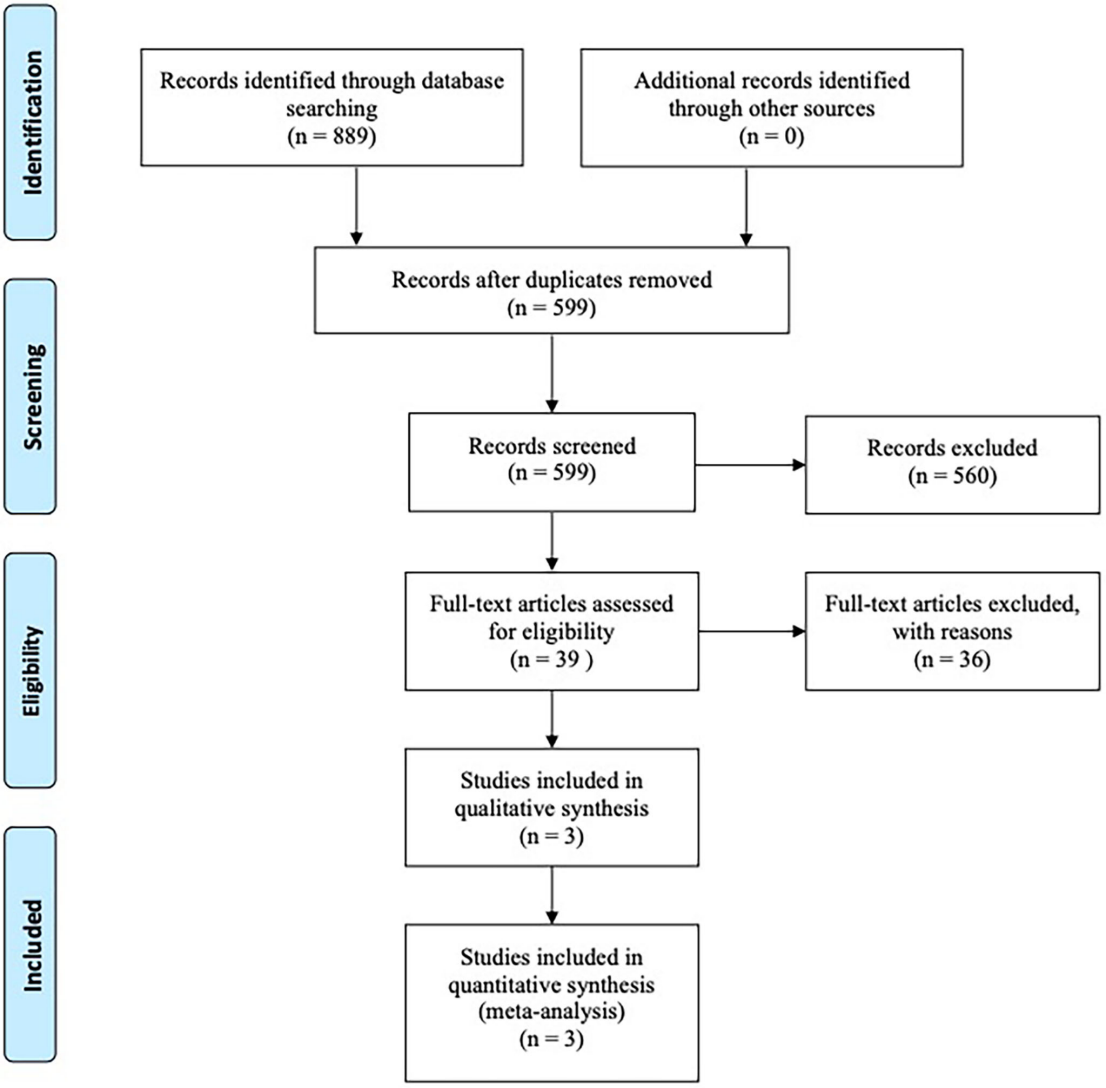
Articles retrieved and assessed for eligibility. After screening process, three RCT articles met the including criteria and were included in ultimate analysis.

Our search strategy initially obtained a total of 889 articles from online databases and other manual sources, of which 190 publications were excluded for duplications. And by screening title and abstract only, 560 articles were excluded for one of the following reasons after meticulous inspection of articles: Not RCTs, Not about ROC, Conference reports, Systematic reviews and Meta-analysis, Case report, Abstract articles review, Single-arm study. After that, we continued to screen the remaining 39 potentially eligible by full text. Three RCTs met the inclusion criteria and were left for further analysis. The selection progress of the included studies is shown in **Figure 1**.

### Characteristics of Included Studies and Patients

Providing available date of PFS and OS for survival, all trials were performed in open-label, phase II setting. These three RCTs assessed one trial with olaparib plus chemotherapy (10), one trial with olaparib and cediranib (11), and one trial with niraparib in combination with bevacizumab (12). There were totally 343 women enrolled for the analysis. The median follow-up ranged from 20.9 to 50.9 months. The main characteristics and results in each trial are listed in **Table 1**.

**Table 1:** Characteristics of studies included in this meta-analysis.

Author year	Phase	Masking	Line	No. of patients	Median age, years	Follow-up, months	Group	Regimen	No. of patients	Median PFS (months)	HR (95% CI) for PFS	Median OS (months)	HR (95% CI) for OS
Oza 2015	II	Open-label	>1	156	60.5 (27-79)	47.7 *vs* 45.7	Intervention arm	Olaparib + Chemotherapy	81	12.2 (9.7 - 15.0)	0.51 (0.34 - 0.77)	33.8 (26.9 - 38.5)	1.17 (0.79 - 1.73)
							Control arm	Chemotherapy	81	9.6 (9.1 - 9.7)		37.6 (27.8 - 44.6)	
Liu 2019	II	Open-label	>1	90	NA	59	Intervention arm	Olaparib + Cediranib	44	16.5 (NA)	0.50 (0.30 - 0.83)	44.2 (NA)	0.64 (0.36 - 1.11)
							Control arm	Olaparib	46	8.2 (NA)		33.3 (NA)	
Mirza 2019	II	Open-label	>1	97	66.5 (58-70)	20.9	Intervention arm	Niraparib + Bevacizumab	48	11.9 (8.5 - 16.7)	0.35 (0.21 - 0.57)	NA	NA
							Control arm	Niraparib	49	5.5 (3.8-6.3)		NA	

NA, not available.

### Assessment of Methodological Bias

The random sequence was generated by using an interactive voice or web response system in all trials. Except for Oza 2015, the remaining trials provided the detailed information about the allocation concealment. None of the trials provided detailed information about the blinding of the participants and personnel. Every trial has low risk detection and attrition bias. Selective reporting only existed in one trial (Liu 2019) for the failure of completely reporting the endpoints originally decided, while other trials offered complete date. And there is no obvious other bias. The assessment methodological bias is shown in **Supplement Figures 1, 2**.

### Efficacy

#### Progression‐Free Survival

**Figure 2:**
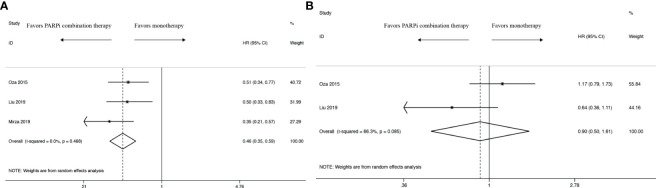
Forest plot of the meta-analysis estimating the hazard ratios and 95% CI of progression-free survival **(A)** and overall survival **(B)** for patients assigned to intervention treatment, compared with those assigned to control treatment.

In PFS analysis, compared with control groups, the pooled HR was 0.46 with 95% CI of 0.35 to 0.59 in the intervention group (**Figure 2A**), which revealed a significantly survival benefit for PFS. In terms of heterogeneity, no heterogeneity was observed (*I* ^2 ^= 0.0%, *P* = 0.468).

#### Overall Survival

Compared with control groups, the pooled HR was 0.90 with 95% CI of 0.50 to 1.61 in the intervention group in OS analysis (**Figure 2B**). There was almost no difference between PARPi combination therapy and monotherapy in OS. Moreover, there existed obviously high heterogeneity *(I^2^ =* 66.3%, P = 0.085).

#### Objective Response Rate

**Figure 3:**
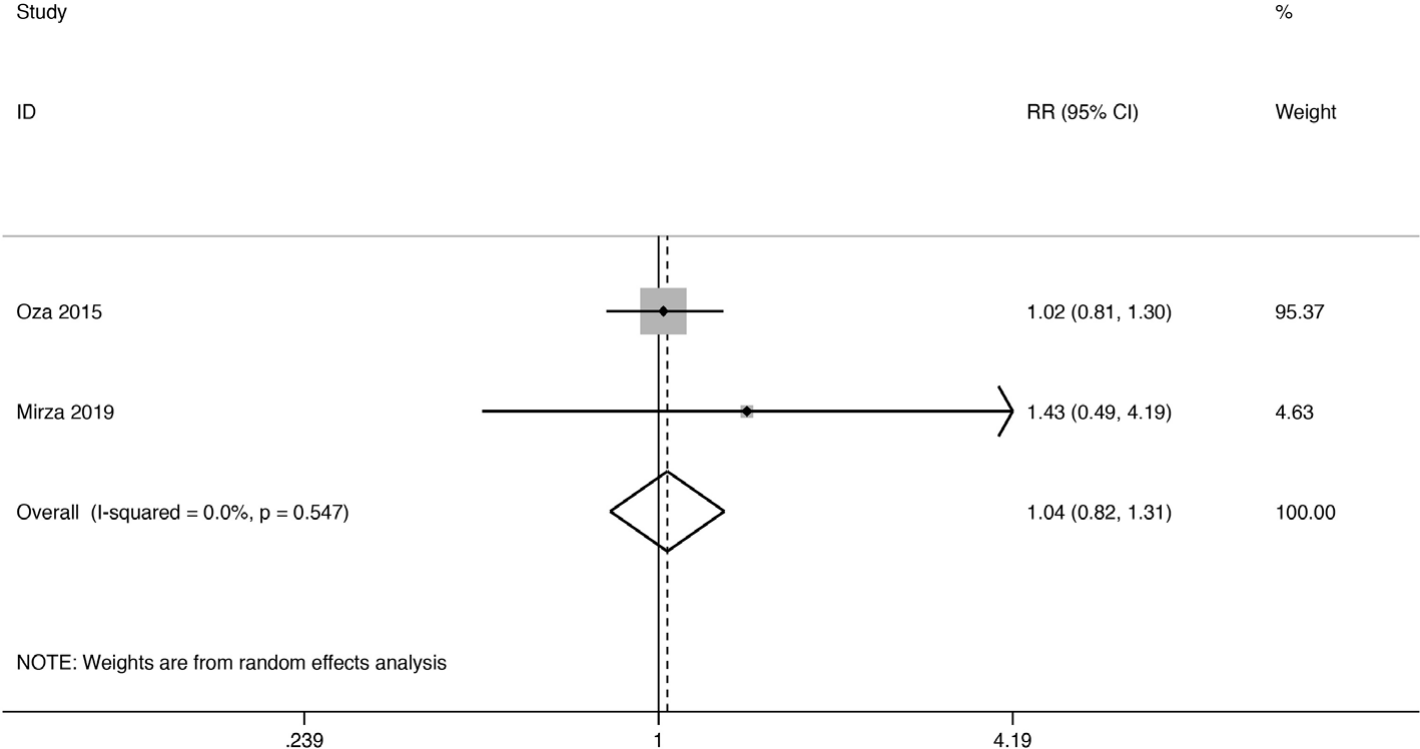
Forest plot of the meta-analysis estimating the relative risk and 95% CI of overall survival for patients assigned to intervention treatment, compared with those assigned to control treatment.

ORR analysis was undertaken both in those women (**Figure 3**). The two RCTs’ provided specific data indicated that PARPi combination therapy didn’t show an ORR advantage over control therapy (RR 1.04; 95% CI 0.82 to 1.31). And no heterogeneity was observed (I^2^ = 0.0%, *P* = 0.548).

### BRCA Status Analysis

**Figure 4:**
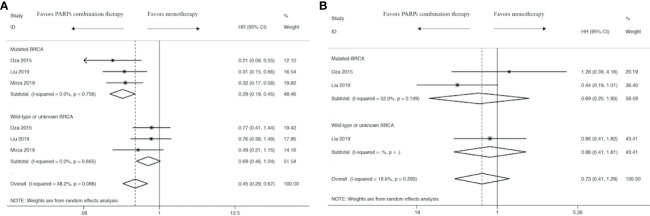
Forest plot of the meta-analysis of subgroup analysis estimating pooled hazard ratios and 95% CI of progression-free survival **(A)** and overall survival **(B)** for patients assigned to intervention treatment, compared with those assigned to control treatment concerning BRCA status.

In order to explore the proper application of PARPi combination therapy, we performed a subgroup analysis concerning HRs in terms of BRCA status. Subgroup analysis showed that BRCA status plays an important role in PFS (**Figure 4A**) and OS (**Figure 4B**) analysis. There was an evident trend to favor PARPi combination therapy over monotherapy in BRCA-mutated women (HR: 0.29, 95% CI: 0.19 to 0.45), while there was no significant difference in women with wild-type or unknown BRCA status (HR: 0.69, 95% CI: 0.46 to 1.04). Meanwhile, the results show that BRCA-mutated women were about the same with BRCA wild-type or unknown women in OS analysis (HR: 0.69, 95% CI: 0.25 to 1.93 *vs* HR: 0.73, 95% CI: 0.41 to 1.29).

### Safety

The RRs of common all-grade and high-grade immune-related AEs are listed in **Table 2**. As for all-grade immune-related AEs, the combination therapy of PARPi and other drugs was relevant with a significantly higher risk of myalgia (RR: 1.67, 95% CI: 1.04 to 2.67), headache (RR: 3.44, 95% CI: 2.05 to 5.27), and diarrhea (RR: 2.85, 95% CI: 1.95 to 4.17). In addition, the risk of thrombocytopenia, anemia, neutropenia, abdominal pain, vomiting, nausea, fatigue was not powered by statistical analysis. Thus, we subsequently performed an analysis to explore the high-grade immune-related AEs in order to identify the danger from the combination therapy. Ultimately, we found it more toxic than control therapy in fatigue (RR: 2.34, 95% CI: 1.12 to 4.89) and diarrhea (RR: 8.32, 95% CI: 1.60 to 43.21). However, the conclusion that combination was more likely to have side effects than control therapy can’t be drawn from remaining immune-related AEs, including thrombocytopenia, anemia, neutropenia, abdominal pain, vomiting, nausea, myalgia, and headache.

**Table 2:** Common treatment-related adverse events in this meta-analysis.

Adverse events	RR (95% CI)	
	All Grade	Grade ≥3
Nausea	1.14 (0.97, 1.33)	1.33 (0.30, 5.85)
Fatigue	1.20 (1.00, 1.44)	2.34 (1.12, 4.89)
Diarrhea	2.85 (1.95, 4.17)	8.32 (1.60, 43.21)
Headache	3.44 (2.06, 5.76)	5.17 (0.62, 43.33)
Myalgia	1.67 (1.04, 2.67)	——
Vomiting	1.32 (0.90, 1.95)	3.10 (0.33, 29.25)
Abdominal pain	1.53 (0.92, 2.53)	1.20 (0.38, 3.80)
Neutropenia	1.17 (0.85, 1.61)	1.27 (0.88, 1.82)
Anemia	1.07 (0.77, 1.50)	0.92 (0.47, 1.81)
Thrombocytopenia	1.28 (0.83, 1.98)	0.81 (0.36, 1.80)

### Sensitivity Analysis

To estimate the influence of single study on overall results of meta-analysis, we conducted sensitivity analysis as presented in **Supplementary Figure 3**; the analysis showed that the pooled results were not significantly changed after deleting each trial, which confirmed the rationality and reliability of our meta-analysis.

## Discussion

To the best of our knowledge, this is the first meta-analysis that compared the efficacy and safety of PARPis in combination with monotherapy in platinum-sensitive ROC patients. In a previous meta-analysis performed by Tomao and colleagues (13), they only compared the efficacy of PARPi monotherapy, while another meta-analysis (14) didn’t stratify results by performing subgroup analysis in different categories: monotherapy and combination therapy, which leads to the loss of the opportunity to better assess the efficacy of PAPRi combination compared with monotherapy. After the three studies included are analyzed, our meta-analysis with pooled results revealed that PARPi combination therapy had a superior PFS to monotherapy in ITT and mutated BRCA population with unsubstantially increased AEs, while OS and ORR couldn’t show benefit from PARP inhibitor combination therapy due to insufficient data.

Clinically proven, the response of patients with ROC is being reduced in the wake of each subsequent line of therapy, especially within platinum-resistant setting. The platinum-based chemotherapy has been recognized as the current standard for ROC with the highest treatment efficacy. And nowadays, neoadjuvant chemotherapy (NACT) is also popularly chosen to be applied in clinical therapy. NACT followed by interval debulking surgery (IDS) and adjuvant chemotherapy (NACT-IDS) and PARPi combination therapy are both popular among the treatment options for ROC. It has been proven that people who carry germline mutations in BRCA1 or BRCA2 genes (15) are more likely to respond to PARPi and chemotherapy combinations. There are also clinical data showing the gene named RAD51 (16) is associated with prolonged OS in people with OC receiving NACT-IDS. The gene status could be a useful key to identify patients more likely to benefit from NACT-IDS or PARPi combinations. And the choice of treatment plan may also be related to the patient’s physical condition and tolerance to therapy. However, precise patient selection criteria to guide therapeutic decisions and the consensus about how to best select them are currently lacking.

The treatment options for patients with ROC described in the ASCO in 2019 said single PARPi or combined with an antiangiogenic agent have confirmed efficacy in prolonging survival time, particularly in progression and recurrence. Thus, there is a recognition that we may benefit more from “getting rid of chemotherapy” than platinum-based chemotherapy. As regards to “get rid of chemotherapy,” previously, Ledermann and colleagues reported results from a randomized phase II study in which simple Olaparib used as maintenance therapy was relevant with an improvement in PFS in patients who suffered platinum-sensitive ROC. On the basis of these findings, the Chinese Medical Association Gynecologic Oncology Branch has developed guidelines to standardize the use of PARPis but focused mainly on first-line maintenance and subsequent therapy as several clinical trials are carried out, such as Study10/19/42, Ariel2/3, NOVA, SOLO-1/2, Quadra, etc. Besides, PARPi monotherapy that relies on the synthetic lethality might be less effective based on single-target mainly because tumorigenesis is a multistep, multistage process related to multiple genes. Therefore, the growing emphasis on combined strategies involving PARPis might place more responsibility on the treatment of ROC patients, and currently being researched. Most significantly, it is in dire need of urgent investigation to explore additional tumorigenic pathways that are expected to increase the efficacy of PARP inhibition.

Moreover, putting the full lifetime of women and disease into consideration, it is of vital importance to draw close attention to the “cost-benefit ratio,” and we should weigh the survival benefits and satisfactory safety when using PARPis in combination. Thus, questions as follows remain to be answered.

Firstly, it’s indispensable to define optimal regimens of drugs of PARPi combination therapy including the exact dosage and combination-type PARPis (niraparib (17), olaparib (18), and rucaparib (19) have been approved by the FDA for platinum-sensitive ROC with exact dose and maintenance during time). But there are no exact guidelines for the use of PARPis in combination therapy for women with ROC. PARPis can be used in combination with chemotherapy, targeted agents, or immune-oncology agents in recent clinical trials. PARPi combinations with chemotherapy (cyclophosphamide, carboplatin, paclitaxel) have been tried in many trials. In a phase II trial that combined carboplatin plus paclitaxel with olaparib, the PFS and OS benefits occurred despite the lower carboplatin dose in the olaparib plus chemotherapy group (20). It indicated olaparib might provide an additive effect or potentiate the cytotoxic effect of the lower carboplatin dose. What’s more, olaparib has been the most extensively tested one with satisfactory results (18). Olaparib has been approved at 300 mg twice daily by FDA, but the dosage of it was up to 400 mg twice daily in various trials (21). Though no significant survival benefits difference was shown between different dosages (22), the incidence of AEs showed a dose-response relationship, for their RR increased with the increase of dosage due to overlapping toxicities (23). But we still need an in-depth study of other drugs for further utilization. As for targeted agents, PARPi combinations with vascular endothelial growth factor (VEGF)-targeted agents are also frequently studied. It was proven that inhibiting VEGF factor (VEGFF) would lead to increased DNA damage and, thereby, increase susceptibility to the effects of PARP inhibition. A phase II study (11) confirmed combination cediranib/olaparib significantly improved PFS and OS compared with olaparib monotherapy. In NSGO-AVANOVA2/ENGOT-ov24 (24), niraparib plus bevacizumab significantly improved PFS *versus* niraparib. The results of immune checkpoint blockade (CPB) monotherapy in OC was rather disappointing (25), while PARPi was found to be capable of enhancing the efficacy of CPB agents *via* coordinating activation of robust local and systemic antitumor immune responses and improving ORR as well (7), which render them a favorable partner to immune CPB. Though PARPis in various combinations with immune CPB including anti-PD-1 (nivolumab, pembrolizumab) or PD-L1 (durvalumab, atezolizumab, avelumab) or CTLA-4 (ipilimumab, tremelimumab) antibodies are being evaluated, clinical trials of combination PARPi and anti-PD-1/PD-L1 get the most encouraging results (26). In the MEDIOLA trial (27), patients were treated with olaparib plus durvalumab and demonstrated partial response (PR) in 17 (53%) and complete response (CR) in 6 (19%). And in the phase I/II TOPACIO trial (28), niraparib and pembrolizumab were used with PR in only 8 (13%) and CR in 3 (5%). And considering data gained from PARPis trials, the efficacy and the tolerance of PARP inhibition decrease with increasing chemotherapy lines, which indicates that earlier utilization of PARPis in ROC treatment may be more beneficial (29). Moreover, apart from the drugs mentioned above, the combination of PARPis with additional drugs that inhibit homologous recombination (HR) has also been proposed. A phase I trial evaluated the combination of olaparib plus alpelisib (30) in patients with OC, which is a PI3K-inhibitor. The demonstrated ORR was 36%, and the patients were mostly platinum-resistant. Another trial (NCT02208375) also evaluated two different olaparib-containing PI3K combinations with ROC. They are mTOR inhibitor vistusertib (AZD2014) and AKT inhibitor capivasertib (AZD 5363). The ORR of the AZD2014 arm was 20%. Since the purpose of the combined application is to reduce overlapping toxicities and ensure clinical efficacy, we should also pay more attention to the differences in clinical efficacy and safety induced by the changes of drug dosage, use cycle, and taking mode. As was shown in a trial, they compared the efficacy and safety of dose modification of olaparib and found that 300 mg b.i.d. tablet was statistically superior to the 200 mg b.i.d. tablet in terms of PFS with unsubstantially increased AEs (31).

What’s more, with a focus on PFS, our meta-analysis confirmed that women with BRCA mutations benefited most, while women with wild-type or unknown BRCA status got no statistically significant result. But two recent meta-analysis showed that PARPis benefited OC patients regardless of their BRCA mutational status (13, 32). It indicated that the inhibition of PARP by PARPi can effectively cause cell death *via* “synthetic lethality,” especially for BRCA-mutated tumor cell because of BRCA gene’s (33) and PARP’s (8, 34, 35) having much to do with DNA repair, which can explain BRCA-mutated women’s better prognosis. But albeit with minor efficacy, wild-type/unknown BRCA status women can still respond to conventional maintenance strategies due to PARPi (36). We have summarized in a table the ongoing combination trials of PARPis (**Table 3**), and we hope the following trials could provide more available date to highlight the value of combination.

**Table 3:** Overview of ongoing clinical trials of PARPis in combinations in recurrent ovarian cancer therapy.

Study identifier	Phase	No. of patients	Intervention model	Group	Regimen	Primary endpoint	Study completion time
NCT03278717	III	618	Parallel	Intervention arm	Olaparib + Cediranib	PFS + OS	December 2023
				Control arm	Olaparib		
NCT04734665	II	44	Single group	Intervention arm	Niraparib + Bevacizumab	PFS + OS	March 2024
				Control arm	/		
NCT04566952	II	68	Single group	Intervention arm	Olaparib + Anlotinib	PFS	October 2023
				Control arm	/		
NCT04149145	II	40	Single group	Intervention arm	Niraparib + M4344	ORR	December 2027
				Control arm	/		
NCT02571725	II	50	Single group	Intervention arm	Olaparib + Tremelimumab	PFS + ORR	July 2027
				Control arm	/		
NCT03579316	II	88	Single group	Intervention arm	Olaparib + Adavosertib	ORR	October 2023
				Control arm	/		
NCT02657889	II	122	Single group	Intervention arm	Niraparib + Pembrolizumab	ORR	July 2021
				Control arm	/		

In terms of safety, by these findings, PARPi combination was relevant with a higher risk of fatigue and diarrhea for high-grade immune-related AEs. Fatigue is probably the most common symptom associated with PARPi treatment. Taking into consideration that PARPi is not only targeted at tumor cells, inhibition of PARP may also contribute to deregulation of normal cells, which may account for PARPi-related fatigue (14). Besides, gastrointestinal epithelial cells also belong to rapidly proliferating cells, whose capacity will be inhibited significantly by PARPi, and may consequently result in diarrhea (37). Dose modification and symptomatic treatment are usually involved to relieve this symptom (38).

Besides, PARPis are also associated with hypercholesterolemia and hypertransaminasemia. It has been found that rucaparib could raise cholesterol levels, but serious AEs of grade 3 or 4 of them are rare (31). So regular monitoring of liver enzymes is suggested when PARPis are used (39). Moreover, PARPis can elevate creatinine concentrations. Swisher et al. reported elevations in creatinine after the usage of rucaparib (40). Elevation in creatinine was also reported within the first few weeks following initiation of rucaparib treatment. That may be caused by the inhibition of some transporters. Rucaparib has been reported to inhibit kidney transporter proteins MATE1 and MATE2-K, which affect the secretion of creatinine consequently (41), while veliparib can also inhibit transporters MATE1 expressed in the liver and transporters OCT2, MATE1, and MATE2-K expressed in the kidney (40).

There are also reports of elevation of aminotransferases ALT and AST in patients treated with niraparib, while olaparib is better tolerated (42). Those AEs are supposed to be associated with myelosuppression (43), and the changes of the types of inhibitors and doses will influence this process (44), because each PARPi has separate chemical structure with diverse off-target effects and vary in different clinical AEs (45).

Differences in the aspects of chemical structure, preclinical potency, and applied doses account for the differences of PARPis (45). As for the chemical structure, they differ in size and rigidity. For example, with two racemic centers, the most rigid and biggest one is talazoparib (46), which has potent trapping activity against PARP1 and PARP2. And the smallest drug is veliparib with molecular mass of 244 (44). That may account for the ranking that talazoparib is the most potent one in trapping PARP while veliparib is the least (47). As for applied doses, different PARPis has been investigated at different doses. For instance, daily dose of talazoparib is only 1 mg, as compared to 300 mg or greater for the remaining PARPis (45), which is influenced by the tolerability of drug use. When it comes to the use of PARPi combinations, myelosuppression is the most noteworthy clinical AE for sometimes it is particularly serious and can be fatal, of which the most common AE is hematological toxicities. The hematological toxicities of niraparib are mainly grade 3 and 4 (48). And there is evidence that platelets and baseline bodyweight are important in dose modifications in patients. Those whose platelet count is less than 15 × 10^4^ cells ml or baseline bodyweight is less than 77 kg may better start with a starting dose of 200 mg daily instead of 300 mg daily, for they are at higher risk of grade 3 or 4 thrombocytopenia. Therefore, people who are taking PARPis should be have detailed blood tests and regular monitoring of blood toxicity performed.

Thus, some matters should be considered to prevent and relieve those symptoms. Firstly, we should assess women’s physical condition and evaluate their tolerability of PARPis based on baseline date before therapy (49). After predicting women at high risk, we should provide regular rigorous monitoring to ensure their safety. Last but not the least, we should resolve side effects in time according to types and severity of individual adverse reactions (50). Referring to the ASCO guidelines (51), based on tolerability and severity, dose modifications and the change of circle in the use management should be taken into consideration.

The strength of our study is that our paper is the first meta-analysis that compared the efficacy and safety of PARPis in combination with monotherapy in platinum-sensitive ROC patients with the result that PARPi combination therapy had a superior PFS to monotherapy in ITT and BRCA-mutated population with unsubstantially increased AEs.

Nonetheless, these results mentioned above must be interpreted with caution, and a great many limitations should be borne in mind. First, the final results might be estimated with a low level of credibility, because data extraction was performed in accordance to study-level evidence rather than individual patient. Secondly, we only included three eligible RCTs covering 343 patients; it is therefore subject to bias and confounding that may have rendered our pooled estimates influenced. Thirdly, given that OS of the study (Mirza 2019) is unavailable and that the included studies are less than three, we failed to delve into subgroup analysis and thus we couldn’t identify the patients who could benefit from PARPis. Besides, since the topic of our paper is PARPi, it is important and vital for us to focus on the BRCA gene, but we didn’t do well in explaining the BRCA gene because of the limited number of trials and insufficiency of survival outcomes so that we fail to figure out whether there is a relationship between the BRCA mutation status and OS. Additionally, not all survival outcomes in our included studies came to quite an ultimate goal. Accordingly, we are unable to obtain a reliable conclusion of pooled OS and ORR because of the high heterogeneity and low robustness in data, for the small sample size and few included studies. Moreover, in the absence of relevant data of AEs based on certain biomarkers like PARP and VEGF inhibitors, we weren’t able to explore the safety profile in terms of BRCA or other potential biomarkers to make clear a specific population who can gain most from PARPi combination therapy. And we do not comment on the situation of dose interruption/delay or discontinuation due to different systemic adverse reactions or conditions and when the medication can be resumed after symptomatic treatment in safety section, which is very important in these studies of monotherapy *versus* combination therapy.

## Conclusion

In general, the data offered by this systematic review and meta-analysis suggested that PARPis likely play a role in the treatment of ROC. In general, PFS appears to be improved in women with ROC. Specifically speaking, BRCA-mutated women received a better PFS benefit in ROC with PARPi combination therapy compared with monotherapy. And our meta-analysis with pooled results also revealed that unsubstantially increased AEs didn’t hinder people benefiting from combination therapy. But in order to find out the better and more efficacious therapy methods regarding optimal drug combinations, appropriate dose of drugs, and patient selection for PARPis, more data are expected from ongoing clinical trials, and the use of PARPis should be encouraged within these studies.

## Data Availability Statement

The original contributions presented in the study are included in the article/**Supplementary Material**. Further inquiries can be directed to the corresponding authors.

## Author Contributions

MY and LS contributed to the design and conception of the study. XD, JY, and NR carried out the collection and processing of data. SG, JY, and LZ performed the data analysis and interpretation. NR and LZ wrote the manuscript. MY and LS revised the manuscript, and MY gave the final approval of manuscript. All authors contributed to the article and approved the submitted version.

## Funding

This study was financially supported by Medical Health Science and Technology Project of Zhejiang Provincial Health Commission (MY, No. 2018KY556); Cultivation Program for Innovative Talent Graduate Students (LZ, No. 721100G00713).

## Conflict of Interest

The authors declare that the research was conducted in the absence of any commercial or financial relationships that could be construed as a potential conflict of interest.

## Publisher’s Note

All claims expressed in this article are solely those of the authors and do not necessarily represent those of their affiliated organizations, or those of the publisher, the editors and the reviewers. Any product that may be evaluated in this article, or claim that may be made by its manufacturer, is not guaranteed or endorsed by the publisher.
