# Supplementary material for: Efficacy and Safety of PARP Inhibitor Combination Therapy in Recurrent Ovarian Cancer: A Systematic Review and Meta-Analysis
Source: Front Oncol. 2021 Aug 13;11:638295. doi: 10.3389/fonc.2021.638295 (PMC8414886; doi:10.3389/fonc.2021.638295)
Supplement: Supplementary file 1 [file DataSheet_1.docx]

***SUPPLEMENTARY MATERIAL***

1. **Supplementary Methods**

**Supplementary Method 1**

1. **Supplementary Figs**

**Supplementary Fig. 1 –** Risk of bias graph

**Supplementary Fig. 2 –** Risk of bias summary

**Supplementary Fig. 3** – Sensitivity analysis from ITT and mutated-BRCA arms

**Supplementary Method 1**

The combined text and medical subject heading (MeSH) terms were cross-searched using MeSH and free word as follows: (ovarian neoplasms [MeSH Terms] OR ovar* [Title/Abstract] AND (neoplas* [Title/Abstract] OR cancer [Title/Abstract] OR malignan* [Title/Abstract] OR tumour* [Title/Abstract] OR adenocarcin* [Title/Abstract] OR carcinom* [Title/Abstract])) AND (Poly(ADP-ribose) Polymerases [MeSH Terms] OR PARP [Title/Abstract] OR Poly ADP Ribose [MeSH Terms] OR ADP-Ribosyl transferase (Polymerizing) [MeSH Terms] OR Poly(ADPR) Polymerase) AND (single [Title/Abstract] OR alone [Title/Abstract] OR combination [Title/Abstract] OR combined with [Title/Abstract] OR plus [Title/Abstract] OR olaparib [Title/Abstract] OR lynparza [Title/Abstract] OR rubraca [Title/Abstract] OR rucaparib [Title/Abstract] OR niraparib [Title/Abstract] OR zejula [Title/Abstract] OR talzenna [Title/Abstract] OR talazoparib [Title/Abstract]) AND (randomized controlled trial [Publication Type] OR controlled clinical trial [Publication Type] OR randomized [Title/Abstract] OR placebo [Title/Abstract] OR drug therapy [MeSH Subheading] OR randomly [Title/Abstract] OR trial [Title/Abstract] OR groups [Title/Abstract] NOT (animals [MeSH Terms] NOT (humans [MeSH Terms])).

**Supplementary Fig. 1 –** Risk of bias graph: Review authors' judgments about each risk of bias item presented as percentages across all included studies.

**Supplementary Fig. 2 –** Risk of bias summary: Review authors’ judgments about each risk of bias item for each included study.

**Supplementary Fig. 3** – Sensitivity analysis from ITT and mutated-BRCA arms: Sensitivity analysis of overall survival from ITT (A) and mutated-BRCA arms (B) in included RCTs.

A


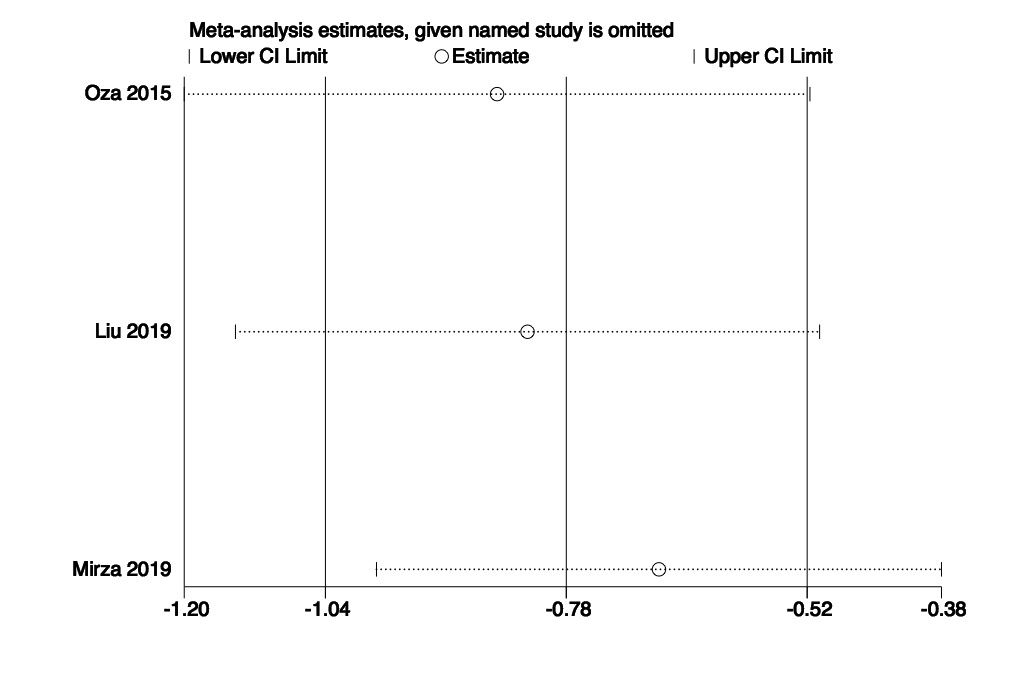


**B
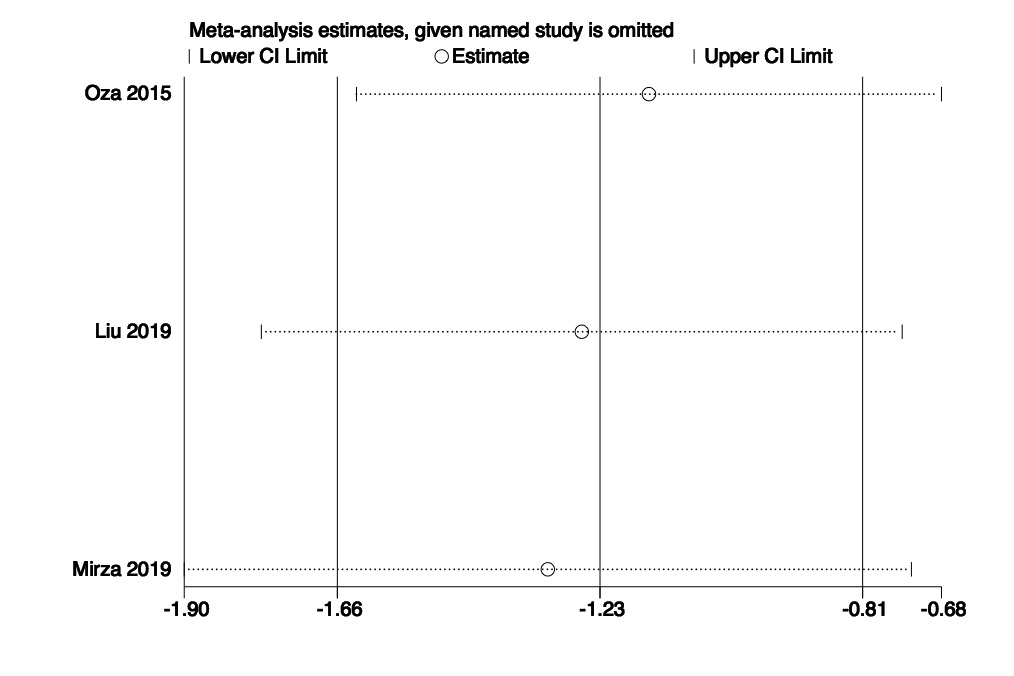
**
